# Views of junior doctors about whether their medical school prepared them well for work: questionnaire surveys

**DOI:** 10.1186/1472-6920-10-78

**Published:** 2010-11-11

**Authors:** Michael J Goldacre, Kathryn Taylor, Trevor W Lambert

**Affiliations:** 1 UK Medical Careers Research Group, Department of Public Health. Oxford University, Oxford, UK

## Abstract

**Background:**

The transition from medical student to junior doctor in postgraduate training is a critical stage in career progression. We report junior doctors' views about the extent to which their medical school prepared them for their work in clinical practice.

**Methods:**

Postal questionnaires were used to survey the medical graduates of 1999, 2000, 2002 and 2005, from all UK medical schools, one year after graduation, and graduates of 2000, 2002 and 2005 three years after graduation. Summary statistics, chi-squared tests, and binary logistic regression were used to analyse the results. The main outcome measure was the level of agreement that medical school had prepared the responder well for work.

**Results:**

Response rate was 63.7% (11610/18216) in year one and 60.2% (8427/13997) in year three. One year after graduation, 36.3% (95% CI: 34.6, 38.0) of 1999/2000 graduates, 50.3% (48.5, 52.2) of 2002 graduates, and 58.2% (56.5, 59.9) of 2005 graduates agreed their medical school had prepared them well. Conversely, in year three agreement fell from 48.9% (47.1, 50.7) to 38.0% (36.0, 40.0) to 28.0% (26.2, 29.7). Combining cohorts at year one, percentages who agreed that they had been well prepared ranged from 82% (95% CI: 79-87) at the medical school with the highest level of agreement to 30% (25-35) at the lowest. At year three the range was 70% to 27%. Ethnicity and sex were partial predictors of doctors' level of agreement; following adjustment for them, substantial differences between schools remained. In years one and three, 30% and 34% of doctors specified that feeling unprepared had been a serious or medium-sized problem for them (only 3% in each year regarded it as serious).

**Conclusions:**

The vast knowledge base of clinical practice makes full preparation impossible. Our statement about feeling prepared is simple yet discriminating and identified some substantial differences between medical schools. Medical schools need feedback from graduates about elements of training that could be improved.

## Background

The broad aims of medical school training are to lay the foundations for a medical career and to provide junior doctors with appropriate knowledge and skills for the first stage of their post-qualification career. Although there is no consensus about how best to train medical students, in the early 1990s it was accepted that medical school training needed to be improved [1-3]. The need for improvement was emphasised by the General Medical Council (GMC) in 1993, with the first publication of *Tomorrow's Doctors*, a directive on undergraduate medical training which stated that 'students must be properly prepared for their first day as a Pre-Registration House Officer'; and revisions in 2003 and 2009 followed [4].

The curricula and student assessment practices of medical schools in the UK have undergone major reforms in recent years. For example, courses on communication skills have been established; and, at some medical schools, problem based learning (PBL) programmes have been introduced [5] to support the development of self-directed learning skills.

In assessing how well medical schools have prepared graduates for work, in addition to relying on judgements by key professional bodies it is important to seek the views of the graduates themselves. Several studies have done so, using postal questionnaire surveys [3,6], interviews [2] and focus groups [7]. Improvements over time have been reported, with doctors who have qualified recently feeling better prepared for work than those in the past [8-11], though some of the studies were limited to a single medical school.

We have reported, nationally across all UK medical schools, on considerable variation between medical schools in how well their graduates felt prepared for the first postgraduate year [3,10]. In this paper, we update and extend our findings. We report, for the first time, on the doctors' views in their third postgraduate year. We covered the third postgraduate year because we considered that the doctors' view of the role of their medical school may have altered with experience. We also, for the first time, report findings by sex, ethnicity and graduate entrant status; report on whether these are confounders in the comparisons between medical schools; report on the broad areas in which respondents did not feel well prepared; and report, for those who did not feel well prepared, on whether this had been a serious, medium-sized or minor problem for them.

## Methods

### Design, setting, and participants

We wrote to all doctors who had qualified from all UK medical schools in 1999, 2000, 2002 and 2005. All four cohorts were surveyed one year following qualification, and the 2000, 2002 and 2005 cohorts were also surveyed at three years. Questions about the transition from medical school, reported here, were contained within questionnaires that were used to study a broader range of subjects including doctors' career intentions, career progression, future plans and views on various topics. Questionnaires were posted to the doctors' registered addresses, provided by the GMC, and supplemented, in year three, by addresses provided by the doctors themselves in year one.

### Questions asked

The following statement was included in the questionnaires: '*Experience at medical school prepared me well for the jobs I have undertaken so far'*. Respondents were invited to state their level of agreement with the statement on a five-point scale from '*strongly agree' *to '*strongly disagree'*. When the 2000 cohort was surveyed one year following qualification, this statement was included in only 25% of the questionnaires (selected at random). In presenting the analysis of the responses to this statement in the surveys at one year, we have combined the responses of the 1999 and 2000 cohorts.

The surveys of the 1999 qualifiers and 2000 qualifiers included just this one question on preparedness. In subsequent surveys we added further questions because we were struck by the low levels of 'feeling well prepared' and by differences in this between graduates of different medical schools. We asked the doctors to indicate in which areas they did not feel well prepared, selecting from '*clinical knowledge', 'clinical procedures', 'administrative tasks', 'interpersonal skills'*, and '*physical/emotional/mental demands'*. We also asked whether not feeling well prepared actually mattered: '*Was lack of preparation a serious, medium-sized or minor problem for you?'*. We invited the doctors to add any 'free text' comments on preparedness, if they wished. These were analysed by theme-scoring the main issues raised.

### Analysis

We used descriptive statistics and χ^2 ^tests to compare responses according to year following qualification (one or three), cohort (1999/2000 combined, 2002, 2005), medical school, sex, ethnicity (white, non-white), whether the doctor had taken an intercalated degree or not, and graduate status at entry to medical school. The entry of these cohorts to medical school largely pre-dated 'fast track' graduate entry and most graduate entrants will probably have had the same medical school experiences as those of the non-graduate entrants. We used binary logistic regression to take account of possible confounding. Binary dependent variables were constructed by, for example, combining respondents who agreed or strongly agreed that medical school had prepared them well, as one group, and all other responses as the second group. In making multiple similar comparisons, we regarded the attainment of a significance threshold of p ≤ 0.01 as evidence of significant difference.

### Ethical approval

The Brighton and Mid Sussex Research Ethics Committee approved the UK Medical Careers Research Group's surveys and studies, in its role as a multi-centre research ethics committee (REC 04/Q1907/48).

## Results

### Response rates

There were 4219 qualifiers in 1999, 4432 in 2000, 4436 in 2002, and 5129 in 2005. Survey response rates were, in the first year, 66% for the 1999s and 2000s combined, 63% for the 2002s and 61% for the 2005s, and in the third year, 67%, 62% and 53% for the 2000s, 2002s and 2005s.

### Feeling prepared for clinical work

At year one, 5.6% of respondents strongly agreed that their medical school had prepared them well for the jobs they had undertaken so far, 42.7% agreed, 20.9% neither agreed nor disagreed, 23.6% disagreed, and 7.2% strongly disagreed.

At year three, the corresponding percentages were 5.0% strongly agreed, 33.8% agreed, 31.6% neither agreed nor disagreed, 23.4% disagreed, and 6.1% strongly disagreed.

We analysed all the data on the five point scale and on a three-point scale combining agree/strongly agree (termed 'agree' in the text and tables that follow) and disagree/strongly disagree (termed 'disagree'). The five-point scale added little. The following descriptions are based on the three-way split.

The percentage of doctors in year one who agreed that they had been well prepared increased from 36.3% (95% CI: 34.6, 38.0) in the cohorts of 1999/2000 to 50.3% (48.5, 52.2) of the 2002s and 58.2% (56.5, 59.9) of the 2005s (Table 1). Those who disagreed fell from 41% to 31% to 21%. Those who strongly disagreed fell from 11.6% to 7.2% to only 2.8%.

**Table 1:** Percentages, by medical school and graduation year, agreeing after one year that they were well prepared

	Year of graduation					
	**1999/2000**		**2002**		**2005**	
			
**Medical school**	**% (95% CI)**	**Rank**	**% (95% CI)**	**Rank**	**% (95% CI)**	**Rank**
						
**5**	73 (64, 82)	1	85 (77, 94)	1	89 (83, 96)	1
**12**	44 (34, 54)	7	64 (53, 75)	5	88 (81, 95)	2
**1**	56 (46, 65)	5	66 (56, 75)	4	78 (70, 86)	3
**9**	20 (12, 28)	23	70 (60, 79)	3	76 (68, 84)	4
**21**	24 (18, 29)	17	70 (63, 77)	2	66 (59, 72)	5
**15**	38 (28, 47)	9	60 (50, 70)	6	64 (55, 73)	6
**18**	35 (26, 44)	10	47 (37, 57)	13	64 (55, 73)	7
**6**	26 (19, 33)	16	43 (35, 50)	15	63 (56, 71)	8
**2**	41 (32, 49)	8	49 (40, 58)	12	62 (55, 70)	9
**11**	59 (50, 69)	3	58 (49, 67)	7	60 (52, 69)	10
**20**	29 (22, 37)	15	44 (36, 52)	14	60 (52, 67)	11
**8**	30 (22, 38)	13	36 (26, 45)	21	60 (51, 68)	12
**7**	23 (16, 29)	19	52 (44, 60)	10	57 (49, 65)	13
**19**	58 (49, 67)	4	39 (29, 48)	18	55 (45, 66)	14
**3**	21 (29, 48)	21	39 (29, 48)	20	54 (44, 64)	15
**22**	24 (17, 32)	18	39 (29, 49)	19	52 (43, 61)	16
**10**	62 (55, 69)	2	56 (48, 63)	8	51 (44, 58)	17
**17**	56 (47, 65)	6	53 (43, 62)	9	49 (39, 60)	18
**23**	30 (23, 36)	14	42 (35, 49)	16	47 (40, 54)	19
**14**	33 (25, 41)	11	50 (41, 60)	11	43 (36, 51)	20
**13**	32 (24, 40)	12	34 (26, 43)	22	42 (34, 51)	21
**4**	20 (12, 29)	22	42 (30, 55)	17	38 (28, 48)	22
**16**	21 (14, 29)	20	33 (24, 41)	23	35 (26, 44)	23

**Total**	36.3(34.6,38.0)	-	50.3(48.5,52.2)	-	58.2(56.5,59.9)	-

The percentage of doctors in year three who agreed that they had been well prepared actually decreased in the successive cohorts: it was 48.9% (95% CI: 47.1, 50.7) for those who qualified in 1999/2000, 38.0% (36.0, 40.0) for the qualifiers of 2002 and 28.0% (26.3, 29.7) for the qualifiers of 2005 (Table 2). Those who disagreed showed no obvious trend with percentages of, respectively, 26%, 33% and 30%.

**Table 2:** Percentages, by medical school and graduation year, agreeing after three years that they were well prepared

	Year of graduation					
	**1999/2000**		**2002**		**2005**	
			
**Medical school**	**% (95% CI)**	**Rank**	**% (95% CI)**	**Rank**	**% (95% CI)**	**Rank**
						
**5**	76 (67, 85)	1	56 (42, 69)	2	73 (62, 85)	1
**12**	54 (42, 66)	7	61 (49, 73)	1	55 (43, 66)	2
**4**	42 (31, 52)	18	38 (25, 51)	10	43 (30, 55)	3
**1**	54 (44, 63)	8	52 (42, 63)	3	40 (29, 50)	4
**2**	47 (38, 57)	14	32 (23, 41)	17	40 (32, 49)	5
**9**	47 (37, 57)	13	52 (41, 63)	4	35 (26, 44)	6
**6**	45 (37, 53)	15	37 (29, 55)	12	32 (23, 40)	7
**11**	69 (60, 78)	2	48 (39, 58)	5	29 (20, 38)	8
**8**	43 (35, 51)	16	29 (18, 39)	20	29 (20, 38)	9
**15**	51 (40, 61)	9	32 (21, 43)	16	28 (19, 37)	10
**13**	39 (31, 47)	22	27 (18, 35)	21	28 (20, 37)	11
**21**	40 (34, 47)	20	45 (37, 53)	7	27 (20, 35)	12
**20**	50 (43, 58)	11	34 (26, 43)	15	27 (19, 34)	13
**3**	57 (47, 67)	5	37 (27, 47)	11	24 (15, 34)	14
**10**	59 (52, 66)	4	35 (27, 43)	13	22 (16, 29)	15
**7**	35 (26, 44)	23	32 (24, 40)	18	22 (14, 30)	16
**19**	64 (54, 74)	3	29 (19, 39)	19	22 (12, 31)	17
**14**	49 (39, 59)	12	44 (34, 54)	9	20 (13, 28)	18
**16**	42 (32, 52)	19	25 (17, 33)	22	19 (9, 29)	19
**22**	42 (33, 51)	17	16 (7, 24)	23	19 (11, 26)	20
**23**	40 (33, 46)	21	35 (27, 43)	14	18 (11, 24)	21
**18**	51 (40, 61)	10	45 (35, 56)	6	17 (10, 25)	22
**17**	55 (46, 64)	6	44 (35, 53)	8	11 (4, 18)	23

**Total**	48.9(47.1,50.7)	-	38.0(36.0,40.0)	-	28.0(26.2,29.7)	-

### Differences between subgroups of doctors

Differences in preparedness by sex, ethnicity, intercalated degree status and graduate entrant status were modest compared to cohort differences. In year one, ethnicity was a statistically significant predictor of doctors' feeling prepared (49.3% of white doctors and 45.3% of non-whites agreed that they felt well prepared, p < 0.001), and sex, intercalated degree status and graduate status at entry to medical school were not (p = 0.05, 0.7, 0.6 respectively). In year three, ethnicity was a statistically significant predictor of doctors' feeling that they had been well prepared (whites 40.4%, non-whites 32.5%, p < 0.001), as was sex (males 41.5%, females 37.0%, p = 0.003) and graduate status (graduate entrants 41.5%, non-graduates 38.1%, p = 0.003). Whether or not the doctor had taken an intercalated degree was not a predictor (p = 0.1). Binary logistic regression modelling (including cohort, medical school, sex, ethnicity, degree status and graduate status) did not materially affect the significant and non-significant results.

### Medical school differences in preparedness

**Figure 1:**
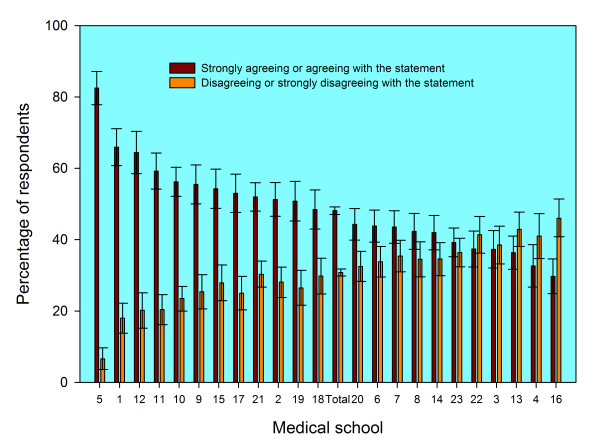
**Responses one year after graduation to the statement "Medical school prepared me well for the jobs I have undertaken so far" from graduates of 23 UK medical schools**. Footnotes: 1. The data shown are aggregated for the UK medical graduates of 1999/2000, 2002, and 2005 surveyed one year after graduation. 2. Error bars denote 95% confidence intervals for the percentages who "Strongly agreed" or "agreed" and who "strongly disagreed" or "disagreed". For each medical school, the percentage of doctors who stated "neither agree nor disagree" is the difference between 100% and the sum of the two percentages shown.

**Figure 2:**
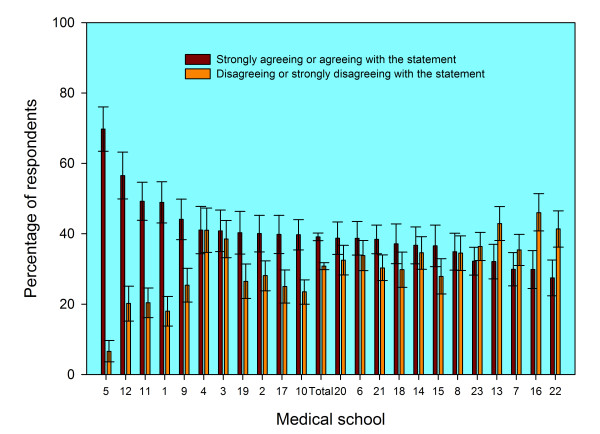
**Responses three years after graduation to the statement "Medical school prepared me well for the jobs I have undertaken so far" from graduates of 23 UK medical schools**. Footnotes: 1. The data shown are aggregated for the UK medical graduates of 1999/2000, 2002, and 2005 surveyed three years after graduation. 2. Error bars denote 95% confidence intervals for the percentages who "Strongly agreed" or "agreed" and who "strongly disagreed" or "disagreed". For each medical school, the percentage of doctors who stated "neither agree nor disagree" is the difference between 100% and the sum of the two percentages shown.

There were substantial differences between graduates from different medical schools in the percentages who agreed and who disagreed that they felt prepared for their first year of work. The numerical codes in the figures and tables denote medical schools, and the same code is used in each figure and table to denote the same school. In year one (Figure 1), the level of agreement varied from 30% at school 16 to 82% at school 5; in year three (Figure 2) it varied from 27% at school 22 to 70% at school 5. In each figure the schools are sorted in declining order of agreement with the statement. The average level of agreement across all medical schools is denoted as 'Total' in the figures, and the 95% confidence intervals may be used to judge significant differences. Changes across cohorts in percentage agreement, and in medical school ranking, are shown in Tables 1 and 2. For some schools, the percentage whose graduates agreed that they were well prepared increased substantial, as did their ranking; for others, relatively low ranking did not change much across cohorts or between years one and three.

Differences between medical schools remained largely unchanged after multivariate adjustment for such factors as sex and ethnicity. In year one, univariate analysis (Figure 1) showed levels of agreement to be significantly high in schools 1, 5, 9, 10, 11 and 12 (based on their confidence intervals not overlapping with that of the overall average). Multivariate binary logistic regression analysis showed the same schools to be significantly high and no others became high (based on analysis of odds ratios, not shown). Schools that were significantly low were 3, 4, 13, 14, 16, 22 and 23; in the multivariate model they all stayed low, and schools 6, 7 and 8 also become significantly low. In year three, Figure 2 shows schools 1, 5, 11 and 12 to be high; the result was the same after multivariate modelling. Schools that were significantly low were 7, 13, 16, 22 and 23; in the multivariate model they all stayed low and 8 also became significantly low.

### Extent to which lack of preparation was a problem

Overall, only 2.9% of responders considered feeling unprepared to be a serious problem (2.5% in year one and 3.3% in year three, p = 0.03). The only significant findings on 'a serious problem' were that, in year one, doctors who had taken an intercalated degree were less likely to specify this (1.8% did so) than those who had not (3.0%, p = 0.004); and in year three, non-whites were more likely to do so (2.8%) than whites 4.1%, p = 0.01). All other differences in years one and three were non-significant.

Lack of preparation was regarded as a serious or medium-sized problem by 31.7% (30.0% in year one and 33.1% in year three, p < 0.001). We merged the serious and medium sized response categories and repeated the analysis. In year one, this confirmed that ethnicity (white or non-white) and medical school were statistically significant predictors (both p < 0.001), independently of other factors, and graduation cohort, sex, intercalated degree status and graduate entrant status were not (p = 0.02, 0.4, 0.05 and 0.5 respectively). In year three, cohort, ethnicity and medical school were statistically significant predictors (all p < 0.001), as was sex (p = 0.02), but not graduate status (p = 0.6) or having an intercalated degree (p = 0.2).

### Areas where respondents did not feel well prepared

The broad areas in which the doctors felt unprepared are shown in Table 3. The category with the highest percentage of 'feeling unprepared' was that of clinical procedures (37.9% of all responses overall) whilst interpersonal skills was the area where the lowest percentage indicated that they did not feel well prepared (3.3% overall).

**Table 3:** Percentage of respondents* who specified that their medical schools had not prepared them well (and denominator numbers, N)

Year 1/characteristic		N	Clinical knowledge	Clinical procedures	Admin	Interpersonal skills	Demands**
							
	**All**	5863	17.9	31.5	33.8	2.8	24.2
							
**Cohort**	**2000**	-	-	-	-	-	-
	**2002**	2750	18.6	36.7^1^	34.9	2.4	26.3^1^
	**2005**	3113	17.3	26.9^1^	32.9	2.1	22.4^1^
							
**Sex**	**Male**	2255	18.1	29.6^1^	37.9^1^	2.8	19.2^1^
	**Female**	3608	17.8	32.7^1^	31.3^1^	1.9	27.3^1^
							
**Ethnicity**	**White**	4206	17.7	30.9	32.9	1.9^1^	23.2^1^
	**Non white**	1604	18.5	32.9	36.2	3.2^1^	26.9^1^
							
**Intercalated**	**Yes**	2481	16.8	31.5	34.3	2.3	23.4
**degree**	**No**	3324	18.9	31.5	33.6	2.2	24.9
							
**Graduate**	**Yes**	603	19.1	30.5	30.5	2.2	24.0
**entrant**	**No**	5222	17.8	31.6	34.2	2.3	24.2
							
							

**Year 3/characteristic**		**N**	**Clinical knowledge**	**Clinical procedures**	**Admin**	**Interpersonal skills**	Demands*
							
	**All**	7795	23.7	42.7	32.4^ns^	4.2	30.7
							
**Cohort**	**2000**	2929	22.3^1^	44.5	33.7^1,ns^	5.0^1^	32.3^1^
	**2002**	2321	22.1^1^	40.8	25.9^1^	3.0^1,ns^	26.2^1,ns^
	**2005**	2545	26.6^1^	42.3	36.8^1^	4.2^1^	33.0^1^
							
**Sex**	**Male**	3107	24.1	40.1^1^	36.5^1,ns^	5.0^1^	24.4^1^
	**Female**	4688	23.4	44.3^1^	29.6^1,ns^	3.6^1^	34.8^1^
							
**Ethnicity**	**White**	5371	24.0	43.1	30.9^1,ns^	3.2^1^	28.9^1^
	**Non white**	1801	22.9	42.4	35.9^1,ns^	6.7^1^	35.5^1^
							
**Intercalated**	**Yes**	2993	22.2^1^	42.2	33.9^1,ns^	4.7	30.6
**degree**	**No**	4160	24.9^1^	43.3	30.8^ns^	3.6	30.3
							
**Graduate**	**Yes**	715	21.1^ns^	39.6	26.2^1,ns^	2.5^ns^	26.7^ns^
**entrant**	**No**	6461	24.0	43.3	32.9^1,ns^	4.2	30.9

* Percentages of respondents who expressed an opinion about the statement 'Experience at medical school prepared me well for jobs I have undertaken so far.' We received replies to these questions from some respondents who had agreed, overall, with the statement on preparedness. We included these replies in our analysis of the specific aspects of feeling unprepared.

** Physical, emotional and mental demands.

^1 ^Significant difference (at 0.01 level) within year 1, or within year 3, for each characteristic

All differences between corresponding cells in year 1 and year 3 were significant, except those marked ^ns^

In all areas, except administration, significantly higher percentages of those in year three than in year one did not agree that they had been well prepared. These differences between year one and year three were found, consistently, in all subgroups of doctors except graduate entrants. At both year one and year three, higher percentages of female doctors than male doctors felt unprepared in the areas of clinical procedures and the demands expected of them, and higher percentages of males than females felt unprepared in administration. Higher percentages of non-whites than whites felt unprepared in the areas of interpersonal skills and demands made of them in both year one and year three, and, in year three, in the area of administration. Higher percentages of non-graduate entrants than graduate entrants felt unprepared in the area of administration in year three.

### Comments made about preparedness

Additional comments were sent by 1891 respondents. 20% of these comments (representing 3.5% of respondents) referred to poor levels of exposure to basic clinical skills and lack of clinical experience. Some made general comments about a lack of 'hands-on' experience or a view that medicine should be taught as 'more of an apprenticeship'. Others referred to a need for more training in specific basic clinical skills, tasks and procedures, including prescribing drugs, acute emergency training, administering warfarin, insulin and fluids, carrying out chest drains, central lines and lumbar punctures, dealing with confused, hypertensive or breathless patients, and using a bleep.

18.9% of those who sent comments (3.3% of all respondents) commented that, in their view, their medical school courses had placed too much emphasis on communication skills and other 'soft' skills, at the expense of clinical teaching, and/or that certain subjects had been taught too little (in particular, basic sciences). Comments about problem based-learning were predominantly adverse. By contrast, a similar percentage indicated that they did not feel fully prepared but did not consider it to be a problem (Additional file 1: Table S1).

## Discussion

### Main findings

Our study shows that, from the doctors' perspective in being prepared for year one, medical school training has improved substantially over time. This supports the findings of others [6-9].

The study indicates that, by year three, presumably in the light of experience, the doctors were less likely to agree than in year one that their medical school had prepared them well. Our findings also show that, in year three, the percentage of doctors who felt that they had been well prepared by their medical school actually declined between the cohorts of 2000 and 2005.

Only 3% of respondents regarded being unprepared as being a serious problem for them. About a third regarded it as a serious or medium-sized problem.

There were significant differences between graduates of different medical schools in their views about being well prepared. These differences remained largely unchanged after adjustment for differences between schools in the demographic characteristics of their graduates. Some differences between schools, in whether they ranked high or low, were sustained across cohorts; others changed, improving their score and ranking, substantially. Thus change is eminently possible.

### Strengths and limitations

Our study is large and national. Our findings are consistent with other contemporary studies at Liverpool [7,8] and Manchester [6] which reported that recent qualifiers feel better prepared than those in the past. Another publication, including some data from our group on the 1999/2000 and 2002 cohorts, reported improvements in preparedness in recent cohorts from UK medical schools [9]. There is also consistency between our study and other studies in reporting concerns about medical school graduates lacking clinical knowledge [6-8]. A recent study shows that the transition from medical school to junior doctor remains stressful in England, with greater levels of clinical experience during the undergraduate years being one of the best mitigators of problems [12].

A further strength is that our group is independent of organisations that employ, or provide training for, or could influence the careers of, the respondents. We believe that we get honest answers.

It is known that doctors' self assessments of ability do not necessarily correlate well with independent assessments of their ability [11]. However, a recent study [13] has reported the concerns of consultants and specialist registrars about some areas of clinical practice in which year one doctors in the NHS were not well prepared. Its findings are consistent with the subjective impressions of the doctors in our study.

We asked about 'interpersonal skills' and found very small percentages who felt these were a problem. Some studies have used other phrases like 'communication skills'. Responders may interpret these phrases in different ways and may consider they cover anything from taking a medical history to breaking bad news to patients [2]. Perhaps, therefore, not much emphasis should be put on our findings on this area.

A high percentage of doctors specified that they neither agreed nor disagreed with the statement that their medical school had prepared them well. Some differences between groups of doctors are more evident in the analysis of 'agree versus other responses' than in 'disagree versus other responses', i.e. some differences are dependent on how the 'neither agree nor disagree' findings are grouped. Where this is so, we are inclined to attach more weight to 'agree versus other responses', on the grounds that doctors who have been well prepared should be able unequivocally to specify that this has been so.

Our study is limited by the possibility of responder bias which we cannot discount or assess. A further limitation of our study is the bluntness of our measure of the extent to which the lack of preparation was a problem. For example, serious concerns about interpersonal skills might have different implications from serious concerns about carrying out a clinical procedure which can readily be learnt.

Our sample sizes are large in this study, and some differences which are shown as statistically significant in the tables are small in percentage terms and hence of limited policy relevance. For example, see the year 3 difference between the 2000 and 2002 cohort in respect of clinical knowledge, or that between whites and non-whites in respect of demands expected of them, both in Table 3.

### Unanswered questions

We have no direct insights into the differences between year one and year three views, but two main reasons for differences are possible. First, by year three, some doctors may not think that their medical school training remains relevant to how well prepared they are in their current practice. They might, accordingly, simply be signalling a view that their preparedness owes more to postgraduate than clinical school experience. Second, speculatively, the findings may reflect a greater recognition, by year three, of mismatches between what they were taught and what they have actually done in practice. The facts that there are sustained differences between graduates of different medical schools, and differences between successive cohorts, suggest that many are answering reflectively about their experience at medical school. It is also noteworthy that differences between years 1 and 3 in their overall responses are corroborated by systematic differences in specific areas of their work (Table 3). This, too, suggests that the doctors are making reflective judgements and not simply regarding the general theme of preparedness as redundant by year 3. One possibility, which we cautioned about in our first study [9], is that any increase in training medical students to be well equipped for the first year of practice, the PRHO/F1 year, should not be at the expense of clinical knowledge of benefit in the longer term. The views of doctors about their training at medical school, expressed beyond the first year after qualification, deserve further exploration.

Our findings can only be interpreted as broad indications of doctors' views and of broad trends. Investigation is needed in much greater depth. Indeed, this is being done, for example, by the GMC (and formerly Postgraduate Medical Education and Training Board) surveys which evaluate different elements of postgraduate training [14].

Another area for further study, highlighted by others [9], is the comparison of subjective feelings of preparedness with independent assessments of doctors' ability. Ethnic minority doctors reported feeling less prepared than white doctors; and in some areas of work, women felt less prepared than men. These differences merit further study: they may reflect differences in learning opportunities, in expectations about how well prepared doctors should be, or in willingness to admit to feeling unprepared. Future research could also investigate the relationship between the medical school curriculum and the graduates' sense of preparedness for practice. A Dutch study suggested that medical school curricula which deliver early clinical experience and foster increasing levels of responsibility appear to produce graduates who feel better prepared [15].

## Conclusions

In training students for the first stage of a medical career, medical schools need to strike a balance between preparing students for their first postgraduate job and for their later years in clinical practice. A balance also needs to be struck between what is taught in medical school, of immediate relevance to the first job, and what is taught as induction at work in the first job. The first year after graduation is the final year of training before doctors become fully registered. It is a period when, under supervision, new doctors put into daily practice the knowledge, skills, behaviours and attitudes that they learned as medical students. It is important to understand more about whether the lack of preparedness is related to the medical school or to the quality of supervision received within the NHS.

The General Medical Council, in the latest edition of Tomorrow's Doctors, is placing emphasis on clinical assistantships and shadowing in the final year of medical school. The medical schools themselves are increasing training in prescribing assessment. With implementation of policies like these, there should be continued improvement. It is also important that the evaluation of changes to medical schools' curricula should not be confined to seeking information from new qualifiers, because medical school training impacts upon the careers of doctors well beyond the first year after qualification.

What should be expected from responses about feeling prepared? Most people starting a new professional job probably will, and probably should, feel unprepared to some extent. The vast knowledge base of clinical practice makes full preparation an impossibility. This is wholly recognised in practice: in the pre-registration year junior doctors work under close supervision from their seniors. Our statement about being prepared, though simple, seems nonetheless to be a discriminating one. For example, it identifies some substantial differences between medical schools and some striking trends in the year one responses. The first year data were collected well into the postgraduate year and therefore represented a reflection on what had been experienced, rather than simply nervous anticipation. We hope that our findings will act as a stimulus to medical teachers to seek feedback from their graduates about what further changes, if any, might be desirable, and practicable, in preparing students for work.

## Competing interests

None. The UK Medical Careers Research Group has no financial relationships with commercial entities that might have an interest in the submitted work; has no spouses, partners, or children with relationships with commercial entities that might have an interest in the submitted work; and has no non-financial interests that may be relevant to the submitted work.

## Authors' contributions

MJG and TWL designed the surveys and this study. KT undertook the analyses, with statistical input from TWL. KT wrote the first draft, all authors contributed to subsequent drafts, and all approved the submitted manuscript. All authors are guarantors.

## Pre-publication history

The pre-publication history for this paper can be accessed here:

http://www.biomedcentral.com/1472-6920/10/78/prepub

## Supplementary Material

Additional file 1 **Examples of comments suggesting the view that, whilst acknowledging not being prepared, it was not considered to be a problem**. The file contains Table S1, which includes illustrative quotations from responders. Click here for file
