# Supplementary material for: Views of junior doctors about whether their medical school prepared them well for work: questionnaire surveys
Source: BMC Med Educ. 2010 Nov 11;10:78. doi: 10.1186/1472-6920-10-78 (PMC3020650; doi:10.1186/1472-6920-10-78)
Supplement: Additional file 1 — Examples of comments suggesting the view that, whilst acknowledging not being prepared, it was not considered to be a problem. The file contains Table S1, which includes illustrative quotations from responders. [file 1472-6920-10-78-S1.DOC]

**Table S1: Examples of comments suggesting the view that, whilst acknowledging not being prepared, it was not considered to be a problem***

| *Being unprepared is inevitable*  Any amount of training as a medical student will probably never prepare you for the real thing.  Being a medical student has no bearing on what happens when you become a doctor as a whole different set of skills need to be developed ….  [Not fully prepared in clinical skills…] but really can only be truly picked up on job.  Difficult until you are working to appreciate what you need to know.  I don't think you can ever be really well prepared!  It's a common rite of passage. Everybody finds the first few weeks difficult.  Same for everyone - thrown in at the deep end!  Only really began to make sense when I had to apply knowledge.  I don't know how you can prepare for this job mentally and emotionally until you are caught up in it and realise the stresses it puts on you. I don't think as a student you are able to appreciate these.  *Not being fully unprepared does not matter*  It's part of developing your personal and social and mental stability.  Unsure how medical school would be able to prepare students for physical & emotional demands of job without putting student off!  Medical school is not the place for addressing emotional and mental demands.  I didn't feel I had enough knowledge about the organisation of the hospital, ways of ordering investigations and necessary paperwork, e.g. discharge/referral letters. However, I may not have concentrated on these if we had been taught them at medical school - they become relevant only when working.  Any lack of experience in admin is very quickly put right in house jobs.  Clinical procedures - learn very quickly on the job. |
| --- |

* Comments such as these were made by 339 respondents (17.9% of comments, 3.2% overall).
